# Vitamin D in Diabetes: Uncovering the Sunshine Hormone’s Role in Glucose Metabolism and Beyond

**DOI:** 10.3390/nu15081997

**Published:** 2023-04-21

**Authors:** Jie Wu, Annette Atkins, Michael Downes, Zong Wei

**Affiliations:** 1 Department of Physiology and Biomedical Engineering, Mayo Clinic Arizona, Scottsdale, AZ 85259, USA; 2 Gene Expression Laboratory, Salk Institute for Biological Studies, La Jolla, CA 92037, USA; 3 Division of Endocrinology, Mayo Clinic Arizona, Scottsdale, AZ 85259, USA

**Keywords:** vitamin D, type 2 diabetes, beta cells, vitamin D receptor, insulin

## Abstract

Over the last decades, epidemiology and functional studies have started to reveal a pivotal role of vitamin D in both type 1 and type 2 diabetes pathogenesis. Acting through the vitamin D receptor (VDR), vitamin D regulates insulin secretion in pancreatic islets and insulin sensitivity in multiple peripheral metabolic organs. In vitro studies and both T1D and T2D animal models showed that vitamin D can improve glucose homeostasis by enhancing insulin secretion, reducing inflammation, reducing autoimmunity, preserving beta cell mass, and sensitizing insulin action. Conversely, vitamin D deficiency has been shown relevant in increasing T1D and T2D incidence. While clinical trials testing the hypothesis that vitamin D improves glycemia in T2D have shown conflicting results, subgroup and meta-analyses support the idea that raising serum vitamin D levels may reduce the progression from prediabetes to T2D. In this review, we summarize current knowledge on the molecular mechanisms of vitamin D in insulin secretion, insulin sensitivity, and immunity, as well as the observational and interventional human studies investigating the use of vitamin D as a treatment for diabetes.

## 1. Introduction

**Figure 1:**
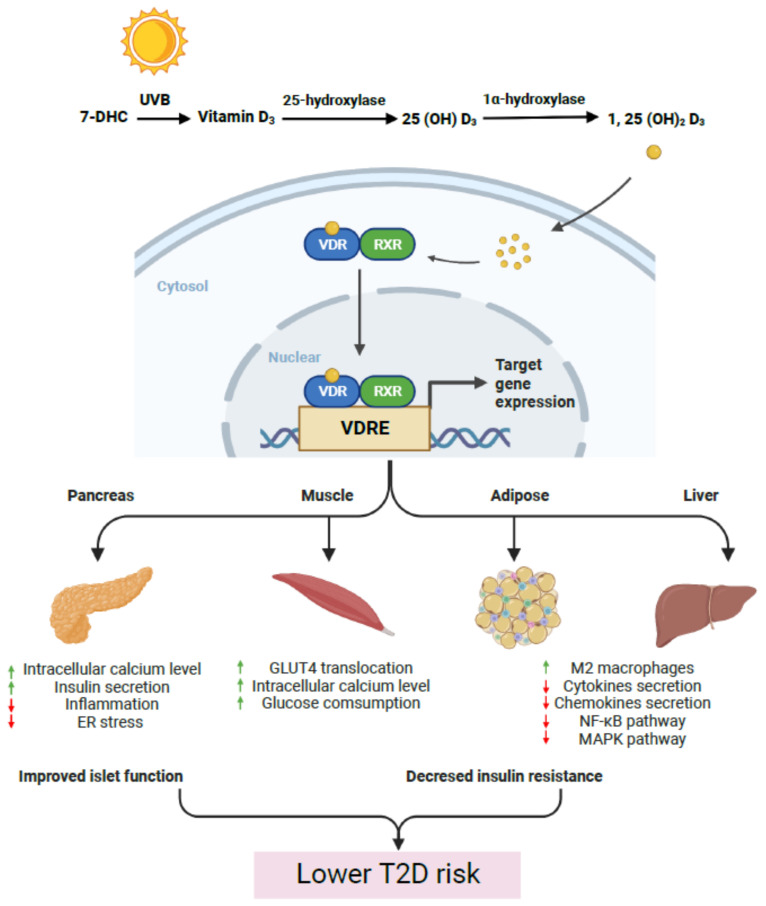
Vitamin D protects against type 2 diabetes. 1,25 (OH)_2_ D_3_, the active form of vitamin D3, is produced from cholesterol through successive hydroxylation of UVB generated 7-dehydrocholesterol (DHC). 1,25 (OH)_2_ D_3_ activates the vitamin D receptor (VDR) retinoid X receptor (RXR) heterodimer in the major metabolic tissues. The active VDR/RXR heterodimer binds to vitamin D response elements (VDREs) to induce changes in gene expression that in combination, improve islet function and decrease insulin resistance.

Vitamin D is a group of fat-soluble secosteroids. This term generally refers to vitamin D_2_ (ergocalciferol), a plant-derived product of sterol ergosterol, and vitamin D_3_ (cholecalciferol), an animal-derived product of 7-dehydrocholesterol. A small quantity of vitamin D, including vitamin D_2_ and vitamin D_3_, can be acquired from dietary sources. The majority of circulating vitamin D, in the form of vitamin D_3_, is formed in the skin from 7-dehydrocholesterol (7-DHC) in the presence of sunlight [1]. Through two successive steps of hydroxylation catalyzed by 25-hydroxylase and 1α-hydroxylase, respectively, vitamin D in humans is progressively converted into 25-hydroxyvitamin D (25(OH)D) in the liver and 1,25-dihydroxyvitamin D (1,25(OH)_2_D) in the kidney (Figure 1) [2]. While 25-hydroxyvitamin D (25(OH)D) is the primary circulating form and an excellent biomarker for overall vitamin D levels [3], 1,25-dihydroxyvitamin D (1,25(OH)_2_D) is the metabolically active form of vitamin D [4].

Vitamin D exerts its effects via both genomic and nongenomic actions. For the genomic pathway, 1,25(OH)_2_D, as a ligand, binds to vitamin D receptor (VDR), a ligand-dependent nuclear receptor that functions as a transcription factor by generating a heterodimer with the retinoid X receptor (RXR) upon ligand binding [5]. The VDR/RXR complex recognizes vitamin D-responsive elements (VDRE), a direct tandem repeat of two hormone response element in the regulatory regions of target genes [5], activating or repressing gene expression in a context-dependent manner (Figure 1). The downstream effects of VDR are tightly regulated by the specific composition of its coregulatory partners, such as chromatin remodelers, co-activators, and co-repressors [6]. Additionally, 1,25(OH)_2_D may bind to membrane-anchored receptors to regulate the activity of signaling molecules or the production of second messengers [7].

In addition to the canonical functions in regulating calcium absorption, bone growth and remodeling [8], vitamin D has other roles in metabolism and immunity. Notably, growing evidence supports that vitamin D plays a relevant role in islet dysfunction and insulin resistance in T2D [9,10,11,12]. From an epidemiology perspective, the worldwide trend of prevalent vitamin D insufficiency [13,14] may be linked to the growing incidence of T2D in humans. We summarize the molecular mechanisms of vitamin D in regulating insulin secretion and insulin action in both homeostasis and T2D, as well as the epidemiology and clinical evidence ascertaining a protective role of vitamin D in T2D pathogenesis. Lastly, we discuss the role of vitamin D in suppressing autoimmunity and preserving islet function in T1D.

## 2. Vitamin D and Islet Dysfunction in T2D Progression

Already a prevalent endocrine disease, the incidence of T2D is expected to escalate rapidly in the coming decades. T2D is characterized by diminished insulin secretion from pancreatic islets and insulin resistance in peripheral organs. Insulin secretion defects and insulin resistance, triggered by chronic and excess nutritional intake, cause glucose intolerance and hyperglycemia. Both β cell mass and glucose-stimulated insulin secretion (GSIS) are reduced even in the early prediabetic stage [15]. The deterioration of β cell function and reduced β cell mass is likely caused by multiple risk factors, including glucotoxicity, lipotoxicity, and elevated inflammation [15,16,17].

β cells express both the vitamin D receptor transcript (*Vdr*) and 1α-hydroxylase (*Cyp27b1*), which catalyzes the activation of 25(OH)D into 1,25(OH)_2_D, consistent with the cell-intrinsic role for VDR [18]. Furthermore, the presence of a VDRE in the human insulin receptor gene promoter region suggests a potential role of vitamin D in influencing insulin action [19], although direct evidence of VDR occupancy at the *INS* locus is still lacking. Several in vivo and ex vivo studies in rats have indicated that vitamin D deficiency in vivo resulted in reduced serum insulin levels and impaired islet insulin secretion in isolated islets [20,21,22]. Conversely, in vitamin D-deficient mice, several studies showed that vitamin D supplementation could restore islet insulin secretion [20,21,22,23,24], suggesting a direct role of vitamin D in regulating islet insulin secretion function. In addition, serum insulin and *Ins2* expression are significantly decreased in VDR-mutant mice [23], suggesting that vitamin D-VDR controls the expression of genes related to insulin expression and secretion.

Evidence corroborating the function of vitamin D in human β cells has been shown in clinical studies in T2D patients [25], prediabetic [26], and non-diabetic populations [27]. However, while the correlation between vitamin D levels and islet function is robust, it should be noted that whether vitamin D treatment can directly improve insulin secretion in humans remains unclear, with intervention clinical trials showing mixed results of vitamin D supplementation in improving human islet function [18,28,29].

Vitamin D regulates insulin synthesis and secretion through multiple mechanisms. On the one hand, the active form of vitamin D, 1,25 (OH)_2_D, binds to VDR and induces genes related to glucose transport, insulin secretion [19], and cellular growth in β cells [30]. On the other hand, vitamin D may indirectly regulate insulin secretion by impacting intracellular calcium concentrations. Calcium triggers insulin release [31] by promoting the mobilization of insulin vesicles and their exocytosis [32]. 1,25 (OH)_2_D leads to depolarization of cytoplasmic membranes in β cells, opening of Ca^2+^ channels and elevation of intracellular Ca^2+^ levels [33,34]. One possible molecular mechanism of this action is that 1,25 (OH)_2_D activates PKA and enhances channel function by phosphorylating L-type voltage-dependent Ca^2+^ channel-related proteins [33]. Moreover, 1,25 (OH)_2_D activates VDR to regulate the expression of voltage-gated calcium channel to enhance insulin secretion [35]. Another mechanism is that 1,25 (OH)_2_D promotes PLC synthesis and activates inositol triphosphate that releases Ca^2+^ from the ER [34,36]. In addition, vitamin D adjusts the expression of calbindin [37,38], a Ca^2+^-binding protein involved in maintaining Ca^2+^ concentrations.

In T2D, islet dysfunction is caused by a combination of stress factors, including glucolipotoxicity, inflammation, ER stress, and Islet Amyloid Polypeptide (IAPP) toxicity. Vitamin D has long been identified as an anti-inflammatory hormone in the immune response. Vitamin D or over-expression of VDR has also been shown to repress cytokine-induced proinflammatory responses and apoptosis in β cell lines and islets [39,40,41]. The inflammation suppressive function of vitamin D is likely because of the direct suppression of NF-κB activation by liganded VDR. In addition to its anti-inflammatory role, vitamin D is also an active suppressor of ER stress and IAPP-induced β cell dysfunction [39]. Vitamin D is able to downregulate essential ER stress players, such as p-PERK, p-IREa, and CHOP in monocytes, liver, and islets [42]. It is unclear, though, whether the suppression is through direct repression of ER stress gene expression or a secondary effect of the anti-inflammatory function of vitamin D.

Although the pleiotropic protective role of vitamin D in islets is clear, vitamin D supplementation showed mixed results in glucose metabolism [1,43,44,45,46]. This may be partly due to the significant reduction in VDR expression in both T1D and T2D islets [41]. A recent elegant mouse study showed that overexpressing VDR in islets was able to rescue the islet dysfunction, suggesting that a supraphysiological activation of VDR may be required to achieve a functional improvement in islet dysfunction [41]. Pharmacologically, we have shown that a combination of vitamin D and BRD9 inhibitors can induce a synergistic activation of the anti-inflammatory response in β cells and protect against islet dysfunction in several T2D mouse models [39]. Mechanistically, we showed that the balance between two antagonizing chromatin remodeling complexes, BRD9-containing BAF, and BRD7-containing PBAF, defined the amplitude and duration of VDR activation [39]. Future dissection of the epigenetic mechanisms regulating VDR activity may provide additional targets to maximize vitamin D signaling potential in reverting islet dysfunction in T2D.

It is noteworthy that the contributions of vitamin D in regulating islet function may also come from non-β endocrine and non-endocrine cells in islets. Islet macrophages express VDR, which suggests that vitamin D may function in islet-resident immune cells [47]. Interestingly, the vitamin D binding protein (DBP, encoded by the GC gene), is highly expressed in dysfunctional α cells and contributes to α cell adaptation [48] and β cell dedifferentiation [49]. Future studies using tissue-specific knockout models will be essential to define the precise function of vitamin D in different islet cell types.

## 3. Vitamin D and Insulin Sensitivity and Resistance

Insulin resistance, defined as an impaired ability of insulin to induce glucose uptake in peripheral tissues resulting in hyperglycemia, is a hallmark of prediabetes and T2D. Vitamin D has been suggested to regulate insulin sensitivity in cell lines and peripheral metabolic organs [43]. Several in vitro studies showed that 1,25(OH)_2_D activates VDR to increase insulin receptor expression [19,50,51], which could subsequently increase insulin sensitivity. Dunlop et al. showed that peroxisome proliferator-activated receptor (PPAR) δ was the primary 1,25(OH)_2_D activated target in several cancer cell lines [52], while subsequent studies suggested an association between PPAR δ and insulin sensitivity through 1,25(OH)_2_D [53,54]. More recent studies have started dissecting the tissue-specific role of vitamin D in insulin resistance. Skeletal muscle is a major organ contributing to insulin resistance. Zhou et al. concluded that 1,25(OH)_2_D ameliorated insulin resistance in C2C12 myotube cells triggered by free fatty acid [55]. Manna et al. demonstrated that 1,25(OH)_2_D enhanced glucose uptake via the SIRT1/IRS1/GLUT4 axis by activating SIRT1, phosphorylating IRS1, and ultimately translocating GLUT4 in myotubes [10]. Moreover, activation of VDR increases Ca^2+^ concentrations in muscle, enhances the translocation of GLUT4, and increases glucose uptake [56]. Together, these results indicate a protective role of vitamin D against insulin resistance in skeletal muscle. In liver and adipose tissue, whether vitamin D directly regulates insulin receptor expression remains unclear. The reduction in insulin receptor gene expression in the livers of diabetic Wistar rats could be rescued with the treatment of vitamin D3 [57]. A different conclusion from high-fat diet-fed mice, however, indicated that vitamin D did not influence the transcript level of the insulin receptor gene in the liver [58]. In contrast, the anti-inflammatory function of vitamin D in liver and adipose is more verified. A recent study showed that activation of VDR acts on resident liver macrophages to reduce liver inflammation and insulin resistance in diet-induced obese mice [59]. Some evidence from VDR macrophage knockout mice supports the beneficial role of vitamin D by showing that deletion of VDR promotes insulin resistance in liver [60]. In obese adipose tissue, vitamin D downregulates the expression of proinflammatory cytokines (IL-1β, IL-6, TNF-α) [61,62] and chemokines (CCL2, CCL5, CXCL10, CXCL11) [63] released by adipocytes and resident immune cells [64], to consequently repress inflammatory responses. A study in human monocytes suggested that the mechanism of downregulation might involve a reduction in transcript and protein levels of TLR2 and TLR4 via VDR [65]. The anti-inflammatory activity of vitamin D partly relies on the suppression of NF-κB and MAPK signaling by VDR [66,67], which binds to and activates MAPK phosphatase-1 [68] and IKBa [39,69]. Moreover, the vitamin D/VDR axis also inhibits monocyte recruitment into adipose tissue and promotes a shift to anti-inflammatory M2 macrophages in adipose tissue [70].

A number of studies have shown that vitamin D is involved in lipid metabolism by regulating adipogenesis, lipolysis, and lipogenesis. The exact function of vitamin D in these processes is likely to be context-dependent. For in vitro studies using mesenchymal cells (MSCs) from adipose tissue or bone marrow, vitamin D promotes differentiation of the adipocyte progenitors, likely through upregulating lineage factors, such as PPARγ and AP2 [71,72,73,74]. In human MSCs, supplementing vitamin D can promote terminal differentiation by increasing the expression of adipogenesis regulators, such as PPARγ and AP2 and functional enzymes, such as LPL [73]. However, these results are contradictory to the fact that the MSCs from VDR whole-body knockout mice also showed an increase in PPARγ and AP2, and an enhancement of differentiation [75]. A similar trend is observed in adipocyte differentiation using glucocorticoids or thiazolidinedione (TZD) [76]. Moreover, in 3T3-L1 cells, a widely used adipocyte progenitor cell line, vitamin D suppresses lipid deposition and terminal differentiation [77,78]. Since most of these studies were performed on in vitro cultured primary cells or cell lines, the various culture conditions could be a major confounding factor.

Several animal models have been used to interrogate vitamin D’s role in adipose tissue function and lipid homeostasis. Mice fed with vitamin D3-containing diet for 3 weeks showed an increase in subcutaneous and visceral fat [79], while mice administrated with calcitriol through a continuous pump showed reduced adipose weight [80]. Mice with whole body knockout of VDR showed a reduced white adipose tissue mass, reduced serum triglyceride, and cholesterol [81,82,83]. Interestingly, the UCP1 expression is significantly elevated in the WAT of these mice [81], suggesting that elevated energy production could be a cause for the reduced WAT mass. Mice with adipose-specific deletion of VDR, on the other hand, have increased visceral fat in females but not in males [84]. Interestingly, the adipose specific knockout of VDR does not change the glucose tolerance [84], suggesting a limited impact of adipose vitamin D signaling on glucose homeostasis.

In addition to the mechanisms discussed above, the pleiotropic role of vitamin D/VDR in insulin resistance may involve (1) vitamin D-induced increases in parathyroid hormone (PTH), which reduces insulin resistance by increasing the quantity of GLUT1 and GLUT4 in vitamin D-deficient adipose tissue, liver, and muscle [85,86]; (2) suppression of the renin-angiotensin-aldosterone system (RAS) activity which impairs β cell function, inhibits peripheral insulin sensitivity [87], hinders GLUT4 recruitment [88], and triggers insulin resistance [89]; (3) a high dose of 1,25(OH)_2_D supplement that can activate the Ca^2+^/CaMKKβ/AMPK pathway to ameliorate insulin resistance and ER stress [90]; and (4) vitamin D preventing ROS formation, an essential activator of insulin resistance [91].

Several clinical studies also support a protective role of vitamin D in insulin resistance. Chiu et al. performed univariate regression analyses on 126 glucose-tolerant subjects and concluded that patients with hypovitaminosis D have a higher risk of developing insulin resistance [92]. Low plasma 25(OH)D levels are also proposed to be a risk factor for T2D [93,94]. A decrease in insulin resistance and increased insulin secretion has been reported with vitamin D supplementation [18,28,95,96]. However, in a separate study in patients with normal levels of vitamin D, supplementation with 1(OH)D failed to improve glucose homeostasis [97], while ergocalciferol supplementation was reported to increase insulin resistance in three vitamin D-deficient T2D patients [98]. These apparently contradictory findings highlight the need for additional clinical studies.

## 4. Vitamin D Deficiency and Type 2 Diabetes—Results of Observational and Intervention Studies and Meta-Analyses

Vitamin D deficiency is defined as a 25(OH)D level of less than 20 ng/mL, according to established consensus [99]. Vitamin D deficiency has long been associated with islet dysfunction, insulin resistance, and increased T2D incidence [43]. While growing evidence in animal models has illustrated the underlying mechanisms of vitamin D in diabetes pathogenesis, as described above, whether vitamin D supplementation could act as a preventative or interventional therapy for T2D remains unclear, with several studies showing mixed results.

The overall link between vitamin D serum levels and metabolic health has been observed in multiple studies. A cross-sectional study including 10,229 subjects showed a negative association between serum 25(OH)D and BMI during winter months [100]. In a cohort study with 9841 participants and 29 years of follow-up, low plasma 25(OH)D was associated with a higher risk of T2D after adjustment for sex, age, BMI, and other health factors [101]. A similar conclusion has been reported in several studies [102,103,104,105]. In a meta-analysis summarizing 21 prospective studies that included 76,220 subjects and 4996 T2D cases, Song et al. highlighted the monotonical association between higher 25(OH)D levels and lower diabetes risk [106]. An increase of 10 nmol/L in 25(OH)D serum concentration is estimated to correlate with a 4% reduction in the T2D incidence [106]. A positive link between 25(OH)D levels and insulin sensitivity and β cell function has been shown in a Californian study measuring insulin sensitivity index and islet secretion capacity in 126 subjects [100]. However, several other studies showed no significant correlation between vitamin D and insulin levels [107] or T2D incidence [108,109].

Based on epidemiological results, it has been postulated that supplementing vitamin D may ameliorate insulin resistance and enhance glycemic control. A single-center, double-blind, randomized placebo-controlled trial performed on 96 non-diabetic participants suggested a significant beneficial effect of vitamin D3 supplement on peripheral insulin sensitivity compared with placebo after six months [26]. A similar conclusion was drawn in trials performed on overweight, and vitamin D-deficient subjects [110] and subjects with impaired fasting glucose [111]. Improvements were also observed in HOMA-IR [13,112], serum fasting plasma glucose and insulin [112], and body weight [113] in patients with T2D after being treated with vitamin D. Additional trials on females with T2D [28] or with gestational diabetes [114] who were given vitamin D supplements or placebo confirmed the positive role of T2D. In contrast, no differences in insulin resistance were observed when 65 Caucasian men with impaired glucose tolerance received vitamin D supplements [97]. Similarly, in a large, multicenter, randomized clinical trial (D2d), daily supplementation with 4000 IU vitamin D_3_ did not appreciably decrease the risk of diabetes among people with a high risk of T2D [115]. Moreover, increases in fasting insulin levels and insulin resistance were reported in three British Asian patients with non-insulin-dependent diabetes and vitamin D deficiency after three months of vitamin D administration [98]. However, a recent meta-analysis, including this dataset, reaffirmed the beneficial role of vitamin D in non-obese subjects, suggesting that supplementation can promote the reversion from prediabetes to normoglycemia [116]. Hence, whether vitamin D can prevent or revert T2D in humans will still need further research.

## 5. Vitamin D in T1D Progression

Type 1 diabetes (T1D) is caused by the autoimmune destruction of pancreatic β cells, leading to insulin deficiency. The development of T1D is a gradual process of breaking tolerance to autoantigens. β cell-specific autoantigens (such as insulin, proinsulin, and IGRP) are presented by antigen-presenting cells (APCs), triggering cytotoxic T cell responses, which cause β cell damage [117,118]. Several studies in non-obese diabetic (NOD) mice have elucidated that pancreas-infiltrated dendritic cells and macrophages function in presenting islet autoantigens [119,120]. Thereafter, islet antigen-reactive CD4^+^ and CD8^+^ T cells induce β cell damage that consequently potentiates the immune response by releasing more self-antigens [121,122,123,124]. In addition to T cells, autoantibody-producing B cells and innate immune cells also participate in destroying β cells [125,126,127].

Animal models and epidemiological studies strongly support the ability of vitamin D to prevent T1D pathogenesis. In CD-1 mice with diabetes induced by daily intraperitoneal injections of low doses of streptozotocin (STZ), intraperitoneal administration of 1α,25(OH) _2_D_3_ protected the diabetic mice from developing hyperglycemia [128]. Long-term treatment with a high dose of 1,25(OH)_2_D_3_ on NOD mice reduced the incidence of insulitis and hyperglycemia [129,130,131]. In humans, epidemiological studies have shed light on the association between vitamin D intake and T1D incidence. In a birth cohort study, a significant reduction in T1D risk was observed in 10,366 children who received 2000 IU of vitamin D daily [132]. Similarly, maternal intake of vitamin D is relevant to a reduced risk of islet autoimmunity in offspring [133], which is consistent with the conclusion from a more recent case–control study that showed that the lower maternal serum concentration of 25(OH)D during pregnancy is correlated with a higher risk of childhood-onset T1D [134]. Zipitis and colleagues concluded that vitamin D supplementation in early childhood prevented the development of T1D in a meta-analysis-based study [135]. A similar conclusion was generated in a EURODIAB (European Community Concerted Action Programme in Diabetes) subgroup multicenter study [136]. Although data from healthy subjects are promising, there are only limited studies supporting the role of vitamin D in delaying T1D development. Gabbay and colleagues [137] suggested that as an adjunctive therapy with insulin, 2000 IU daily supplementation of vitamin D3 slowed the decline of residual β cell function in patients with new-onset T1D. Two other studies, however, showed that there was no protective effect of 1,25(OH)_2_D_3_ treatment in subjects with new-onset T1D [138,139]. Therefore, more trials evaluating the function of vitamin D supplementation in treating T1D are still needed.

The beneficial effects of vitamin D in T1D could be rooted in its versatile functions in various immune populations. VDR is expressed in nearly all immune cells, including activated T and B cells, dendritic cells, macrophages, and neutrophils [140,141,142,143]. Differentiation of monocytes to macrophages or dendritic cells correlates with a decreased expression of VDR [144,145], whereas T cell activation is accompanied by increased expression of VDR [140,146]. The presence of VDR in both T cells and antigen-presenting cells suggests distinct, cell-type specific mechanisms of vitamin D in suppressing adaptive immunity [147,148]. In monocytes/macrophages, 1,25(OH)_2_D_3_ reduces MHC II and co-stimulatory molecules (CD40, CD80 and CD86) expression and prevents T cell activation [149,150]. In rat and human dendritic cells, 1,25(OH)_2_D_3_ inhibited dendritic cell maturation and stimulatory functions. 1,25(OH)_2_D_3_ treatment inhibits the expression of CD1a^+^ (dendritic cell marker), MHC II, and co-stimulatory genes while maintaining the expression of monocytic markers [151,152,153,154]. 1,25(OH)_2_D_3_ has also been demonstrated to induce dendritic cell apoptosis [151,155], or induce tolerogenic dendritic cells featuring a reduced expression of CD40, CD80, and CD86 [156,157]. Tolerogenic dendritic cells inhibit autoimmune processes by enhancing Treg cell development in NOD mice [156]. Another potential role of 1,25(OH)_2_D_3_ in dendritic cells is to induce the expression of the mannose receptor, the endocytic capacity-related molecule involved in antigen-capturing [158]. Lymphocytes are also profoundly impacted by 1,25(OH)_2_D_3_. Th1 and Th17 cells are essential in T1D initiation [159,160]. 1,25(OH)_2_D_3_ inhibits the expression of multiple cytokines, such as IL-12 and IL-23, and consequently drives a T cell subpopulation shift from Th1/Th17 to Th2 [161,162,163,164]. On the other hand, the recruitment of T cells to the pancreas by cytokines and chemokines aggravates β cell damage. 1,25(OH)_2_D_3_ is able to suppress T cell infiltration by reducing gene expression and/or secretion of multiple cytokines (IL-6, IL-15) and chemokines (CCL2, CCL5, and CXCL10) that manipulate T cell migration [69,165,166]. Furthermore, 1,25(OH)_2_D_3_ suppresses autoreactive T cells and maintains tolerance [167] by promoting Treg cell development [168] and suppressing proinflammatory cytokines (IL-2, IFN-ɤ, IL-17, and IL-21) expression [169]. In addition to its effect on T cells, 1,25(OH)_2_D_3_ also inhibits B cell proliferation, differentiation in memory B cells, and production of immunoglobulins [170]. Whether the action of vitamin D on B cells is required for its T1D prevention capacity remains to be elucidated.

## 6. Conclusions

In addition to its canonical role in skeletal function, vitamin D modulates insulin secretion and action in diabetes. Vitamin D/VDR directly regulates functional genes, including critical genes in the secretion pathway and insulin action. As an anti-inflammatory hormone, vitamin D also acts on tissue resident immune cells to reduce local and systemic inflammation, thus preventing islet, liver, and muscle dysfunction. Though vitamin D is known to work on multiple organs and cell types, the relative contribution of individual cell types to the anti-diabetic effects remain to be determined. Mechanistically, how does vitamin D activate essential functional genes while repressing inflammatory targets? The cell type-specific regulatory circuitry of vitamin D-VDR remains to be elucidated. Vitamin D deficiency is prevalent in the general population and is linked to a higher type 2 diabetes incidence. Normalizing the vitamin D levels in deficient patients has slowed T2D progression. However, large-scale clinical trials have not demonstrated the clinical benefit of vitamin D supplements in ameliorating type 2 diabetes [171]. These results raise more questions for future studies: What is the optimal vitamin D level? Can vitamin D supplements achieve this level without causing side effects? Further larger-scale prospective trials may still be required to test whether vitamin D intake is able to prevent or reverse type 2 diabetes. In T1D, the evidence of vitamin D in preventing at-risk subjects from developing diabetes is lacking. It is also unclear whether the beneficial effects of vitamin D depend on its ability to reprogram autoimmunity, prevent B cell damage, or both.

## Data Availability

Not applicable.
